# Author Correction: Evolutionary divergence of novel open reading frames in cichlids speciation

**DOI:** 10.1038/s41598-021-88187-7

**Published:** 2021-04-14

**Authors:** Shraddha Puntambekar, Rachel Newhouse, Jaime San-Miguel, Ruchi Chauhan, Grégoire Vernaz, Thomas Willis, Matthew T. Wayland, Yagnesh Umrania, Eric A. Miska, Sudhakaran Prabakaran

**Affiliations:** 1 Department of Biology, Indian Institute of Science Education and Research, Pune, Maharashtra 411008 India; 2 Department of Genetics, University of Cambridge, Downing Site, Cambridge, CB2 3EH UK; 3 The Wellcome Trust/CRUK Gurdon Institute, University of Cambridge, Cambridge, CB2 1QN UK; 4 Wellcome Sanger Institute, Wellcome Genome Campus, Cambridge, CB10 1SA UK; 5 Department of Zoology, University of Cambridge, Downing Site, Cambridge, CB2 3EH UK; 6 Cambridge Centre for Proteomics, Department of Biochemistry, University of Cambridge, Tennis Court Road, Cambridge, CB2 1QR UK; 7 St. Edmund’s College, University of Cambridge, Cambridge, CB3 0BN UK

Correction to: *Scientific Reports* 10.1038/s41598-020-78555-0, published online 09 December 2020

The original version of this Article contained a typographical error in the spelling of the author Jaime San-Miguel, which was incorrectly given as Jaime San Miguel Navas. This has now been corrected in the PDF and HTML versions of the Article, and in the accompanying Supplementary Information file.

Furthermore, the Author Contributions section now reads:

“Sh.P. did the transcriptomic, evolutionary and over all data analysis and gave inputs for writing the manuscript. R.N. did the initial transcriptomic and proteogenomic analysis. J.S.-M. did the time-calibration analysis. R.C. did the proteomics experiments. T.W. did the initial quantitative transcriptomic analysis. M.T.W. did the initial transcriptomic analysis. Y.U. participated in the proteogenomics analysis. G.V. and E.A.M. generated the ON and PN liver transcriptomic datasets. S.P. designed and supervised the project, analysed the data, and wrote the manuscript.”

