# Supplementary material for: Evolutionary divergence of novel open reading frames in cichlids speciation
Source: Sci Rep. 2020 Dec 9;10:21570. doi: 10.1038/s41598-020-78555-0 (PMC7726158; doi:10.1038/s41598-020-78555-0)
Supplement: Supplementary file 1 — Supplementary Information. [file 41598_2020_78555_MOESM1_ESM.docx]

**Supplementary Materials**

**Evolutionary divergence of novel open reading frames in cichlids speciation**

Shraddha Puntambekar^1^, Rachel Newhouse^2^, Jaime San-Miguel^2^, Ruchi Chauhan^2^ , Grégoire Vernaz^2,3,,4^, Thomas Willis^2^, Matthew T. Wayland^5^, Yagnesh Urmania^6^, Eric A. Miska^2,6,4^, and Sudhakaran Prabakaran^1,2,7^*

^1^Department of Biology, Indian Institute of Science Education and Research, Pune, Maharashtra, 411008, India

^2^Department of Genetics, University of Cambridge, Downing Site, CB2 3EH, UK

^3^The Wellcome Trust/CRUK Gurdon Institute, University of Cambridge, Cambridge, CB2 1QN, UK

^4^Wellcome Sanger Institute, Wellcome Genome Campus, Cambridge CB10 1SA, UK

^5^Department of Zoology, University of Cambridge, Downing Site, CB2 3EH, UK

^6^Cambridge Centre for Proteomics, Department of Biochemistry, University of Cambridge, Tennis Court Road, Cambridge, CB2 1QR, United Kingdom

^7^St Edmund’s College, University of Cambridge, CB3 0BN, UK

*Corresponding author, email: [sp339@cam.ac.uk](mailto:sp339@cam.ac.uk).

**Supplementary Figure 1**

**
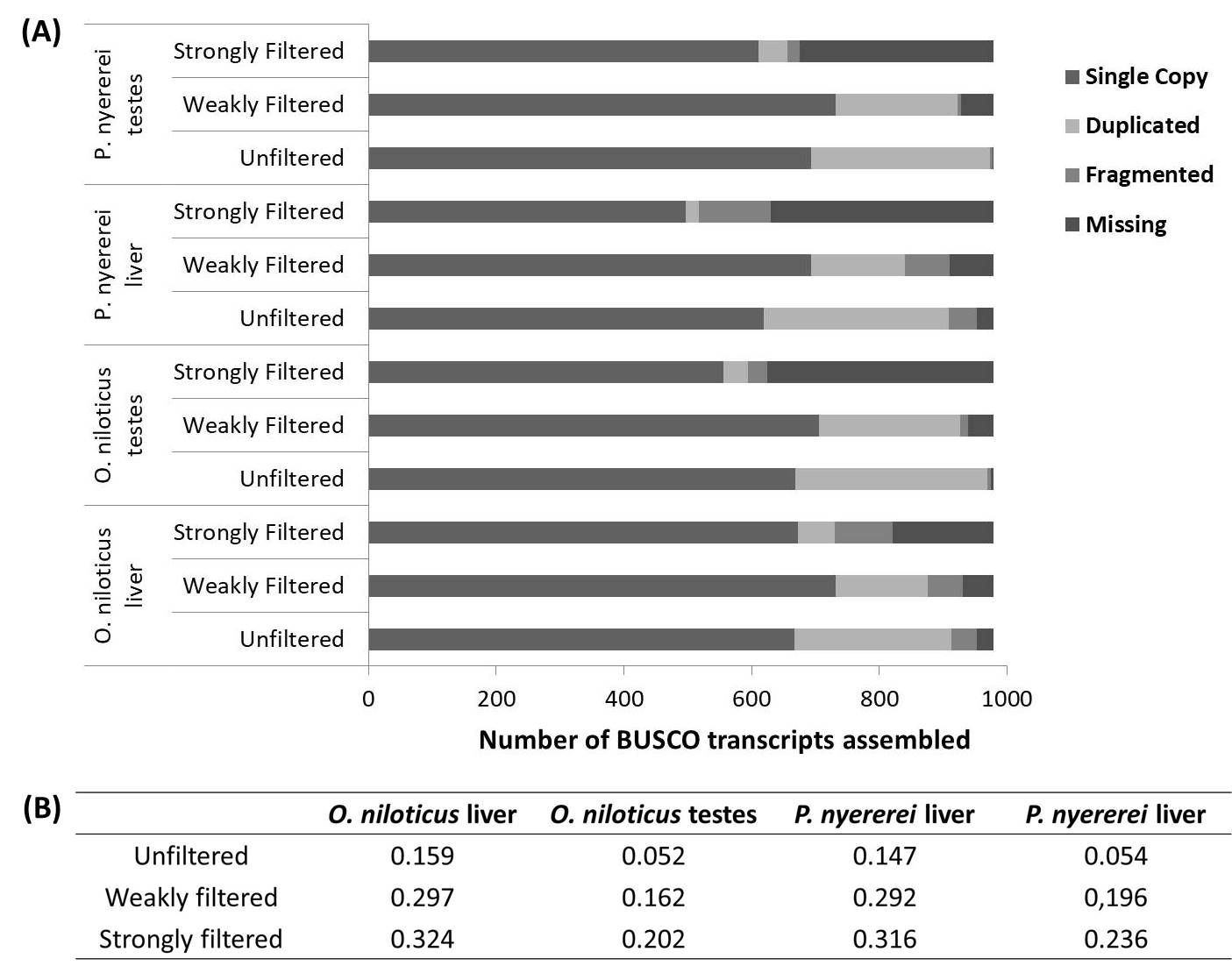
**

**SF. 1. Processing assembled transcripts.**

1. The number of BUSCO metazoan transcripts present in the unfiltered and filtered Trinity transcriptomes. Weakly filtered: transcripts with a Transrate score of 0.01 or lower removed. Strongly filtered: transcript removal threshold set to optimise the overall assembly Transrate score. Dark gray: single copy. Light gray: duplicated. :fragmented. Black: missing.
2. The effects of filtering on the whole assembly Transrate scores for each Trinity transcriptome. Weakly filtered: transcripts with a Transrate score of 0.01 or lower removed. Strongly filtered: transcript removal threshold set to optimise the overall assembly Transrate score.

**Supplementary Figure 2**

**
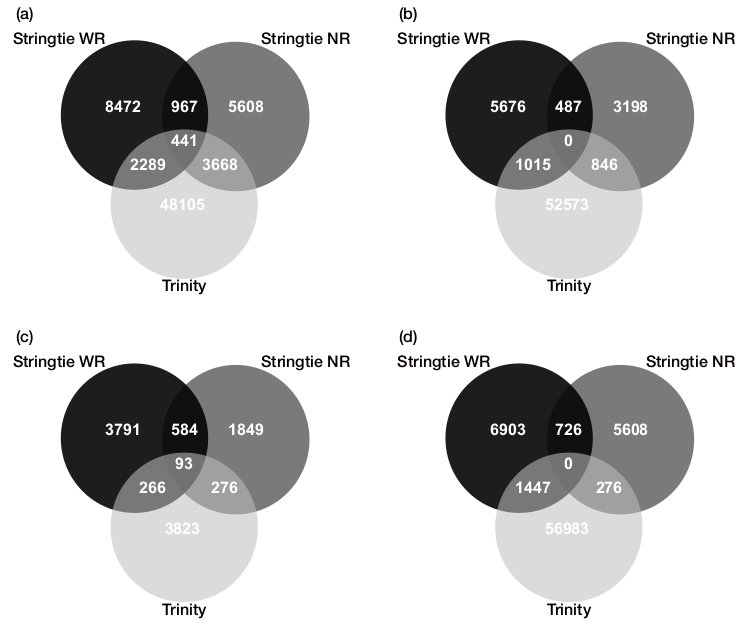
**

**SF 2.** The overlap in species-specific transcripts identified using each transcriptome

assembly method. Species-specific transcripts were identified as those without a match

of at least 80% at the nucleotide level in the equivalent transcriptome in the opposing

species. The transcripts identified by each method were compared using GFFcompare.

(a) *O. niloticus* testes (b) *O. niloticus* liver (c) *P. nyererei* testes (d) *P. nyererei* liver

**Supplementary Figure 3: Functional annotation analysis of species-specific transcripts.**

**
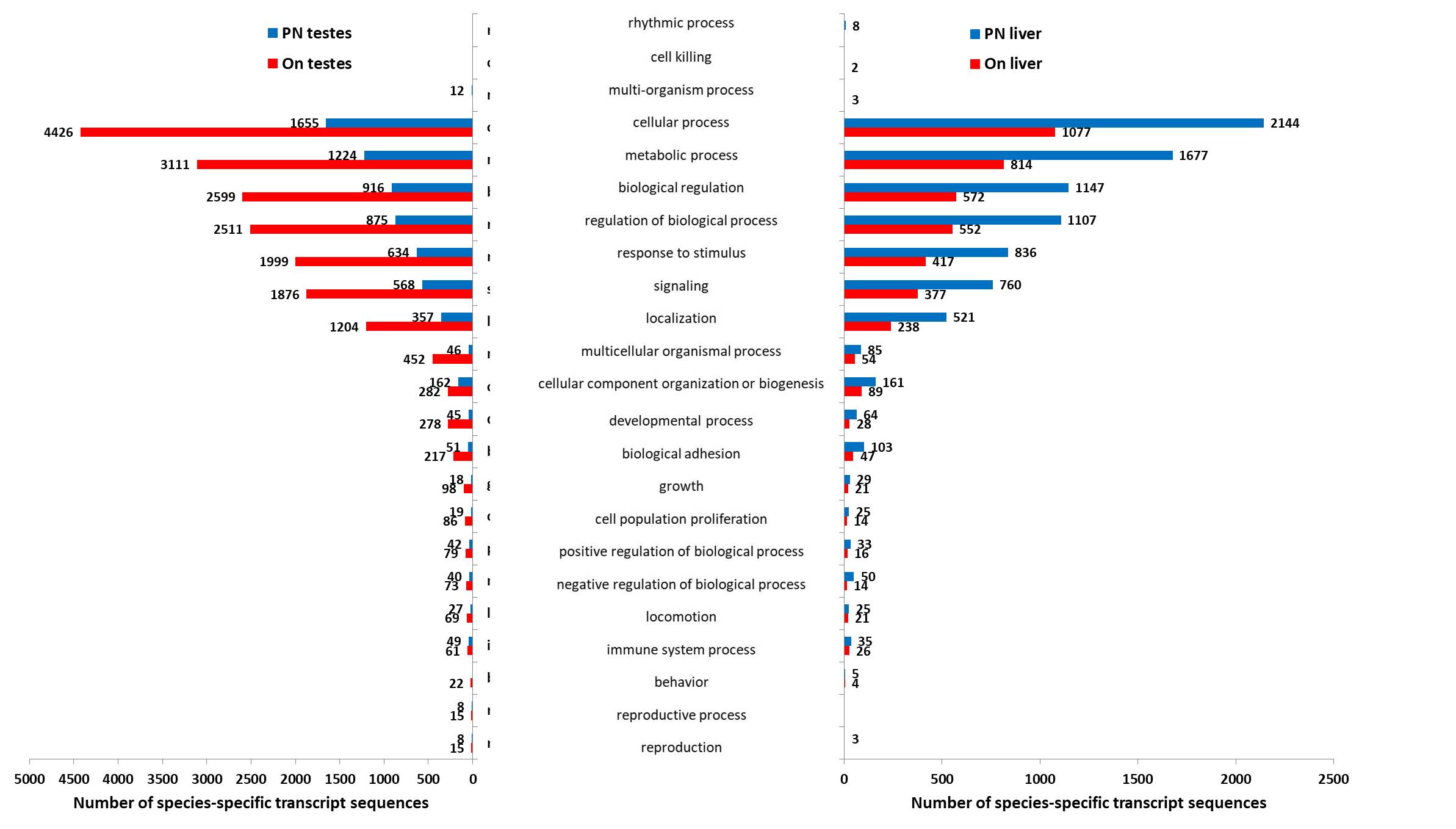
**

**SF. 3: Functional annotation analysis of species-specific transcripts.** The Level 2 Biological Process GO Annotations of Species-Specific transcripts for each species and tissue. The union of the species-specific transcripts identified using each transcriptome assembly method was annotated using InterProScan

**Supplementary Figure 4**

**
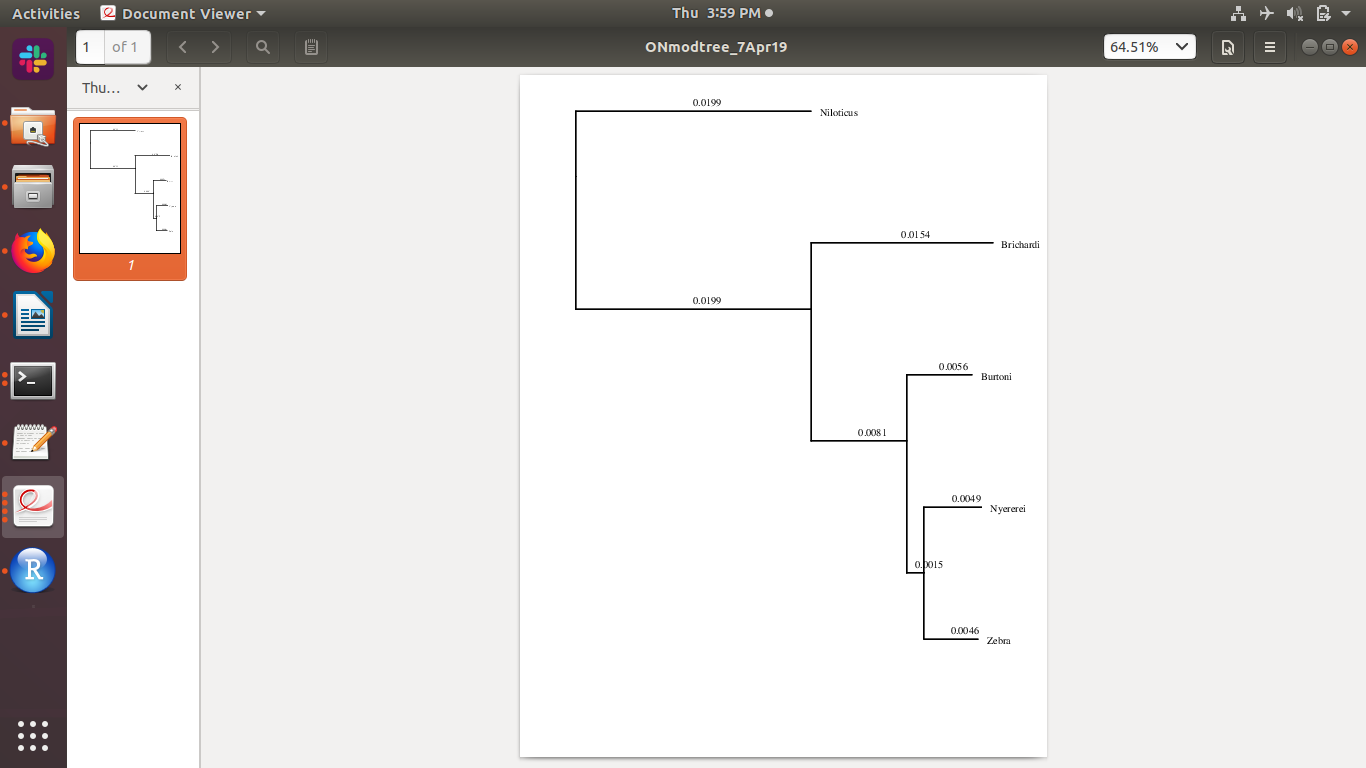
**

**SF. 4 Phylogenetic tree constructed over four-fold degenerate sites from the alignment of five cichlids genome.** The numbers on the edge represent the neutral species divergence calculated by phyloFit.
